# The extracellular matrix in development

**DOI:** 10.1242/dev.175596

**Published:** 2020-05-29

**Authors:** David A. Cruz Walma, Kenneth M. Yamada

**Affiliations:** Cell Biology Section, National Institute of Dental and Craniofacial Research, National Institutes of Health, Bethesda, MD, 20892-4370, USA

**Keywords:** Extracellular matrix, Embryo, Migration, Adhesion, Differentiation, Biophysical

## Abstract

As the crucial non-cellular component of tissues, the extracellular matrix (ECM) provides both physical support and signaling regulation to cells. Some ECM molecules provide a fibrillar environment around cells, while others provide a sheet-like basement membrane scaffold beneath epithelial cells. In this Review, we focus on recent studies investigating the mechanical, biophysical and signaling cues provided to developing tissues by different types of ECM in a variety of developing organisms. In addition, we discuss how the ECM helps to regulate tissue morphology during embryonic development by governing key elements of cell shape, adhesion, migration and differentiation.

## Abstract (precis)

**Summary:** This Review discusses our current understanding of how the extracellular matrix helps guide developing tissues by influencing cell adhesion, migration, shape and differentiation, emphasizing the biophysical cues it provides.

## Introduction

The extracellular matrix (ECM) is essential for metazoan life; without it, we would be merely an amorphous mass of cells. The ECM is the non-cellular component of all tissues, forming the physical environment surrounding cells, and playing both structural and signaling roles (Alberts et al., 2014; Frantz et al., 2010; Hynes and Yamada, 2012; Loganathan et al., 2016). As summarized in this Review, the physical roles of the various types of ECM include anchoring, guiding or restraining cell and tissue movements. For example, epithelial cells are anchored to a basement membrane, but if they become migratory, they can migrate along ECM fibrils or basement membranes.

The physical properties of the ECM (e.g. stiffness) can provide regulatory information to cells (Frantz et al., 2010; Yamada and Sixt, 2019). In addition, the ECM can provide signaling information through its specific biochemical composition and the local concentrations of its constituents (e.g. for gene regulation), and can serve as a reservoir and source of signaling molecules, such as cytokines. The many developmental and cell biological processes regulated or guided by the ECM include: contact guidance-mediated cell directionality (Teixeira et al., 2003), morphogenetic movements of gastrulation and organogenesis (Loganathan et al., 2016; Dzamba and DeSimone, 2018; Wang et al., 2017), stem cell differentiation (Darnell et al., 2018a,b; Smith et al., 2018), anchorage-dependent growth (Huang and Ingber, 1999), and cell survival (*anoikis*) (Frisch and Ruoslahti, 1997; Reddig and Juliano, 2005).

The importance of the ECM in normal mouse and human development is demonstrated by the many examples of embryonic lethality or functional disorders caused by deficiency or mutation, either experimentally or in a multitude of genetic diseases. Human genetic disorders can result from perturbed ECM structure, dynamics, components and/or interactions (Arseni et al., 2018; Bateman et al., 2009; Lamande and Bateman, 2019; Pozzi et al., 2017). In comparison with studies of the ECM in development, the regulation of developmental processes by transcription and growth factor signaling are much better studied (Chen et al., 2018c; Schweisguth and Corson, 2019; Shahbazi and Zernicka-Goetz, 2018). However, new insights highlight the importance of synergy between biochemical and biophysical signaling in developing tissues. As such, interest in the ECM and the biophysical cues that regulate embryogenesis is growing (Mammoto et al., 2012; Merle and Farge, 2018; Dzamba and DeSimone, 2018).

**Fig. 1.**
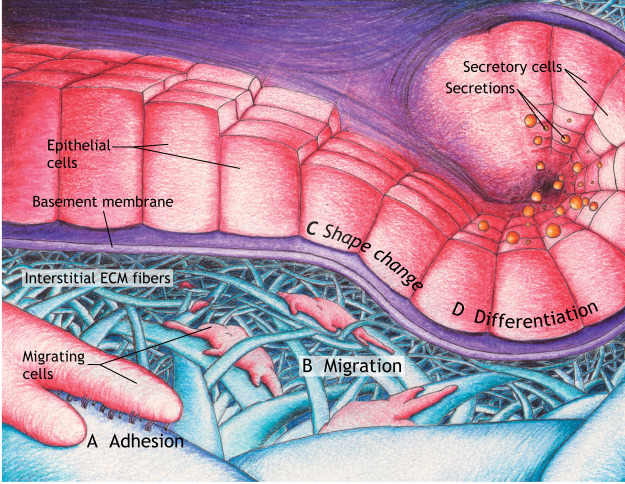
**A summary of the regulation of developmental processes by the extracellular matrix.** Two major forms of ECM are basement membranes and interstitial matrices. These types of ECM help to direct cell and tissue shape during morphogenesis in development by influencing cell adhesion (A), migration (B), morphology (C) and differentiation (D). (A) Cell-ECM adhesion, with cell adhesion complexes between the cell and a fibril substrate shown being mediated by integrins (heterodimeric receptors projecting downward from the closest migrating cell). (B) Migrating cells using oriented protrusions and cell-ECM adhesion complexes to move along interstitial ECM fibers. (C) Epithelial cells undergoing shape change (columnar-to-cuboidal transitions). (D) Epithelial cells differentiating into secretory cells.

This Review summarizes key roles of the ECM in cellular processes and tissue morphogenesis during embryonic development. After first introducing principles of cell-ECM interactions, we focus primarily on recently published examples to discuss how the ECM helps to direct developing tissues by influencing cell adhesion, migration, shape and differentiation (Fig. 1A-D). We place particular emphasis on the biophysical properties and signals of the ECM that regulate these processes in a variety of organisms, ranging from humans and mice to *Drosophila* and *Tribolium*.

## Extracellular matrix

**Fig. 2.**
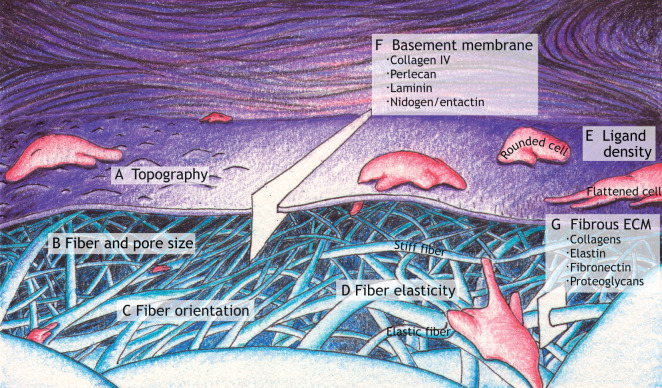
**Examples of physical properties of the extracellular matrix.** (A) Topography encountered by a migrating cell. (B) Examples of varying fiber diameters and sizes of pores between ECM fibers. (C) Examples of fiber orientation: compare oriented fibers near ‘C’ with the other relatively non-oriented fibers. (D) Examples of varying fiber elasticity/stiffness represented as different degrees of fiber deformation as a cell pulls on two fibers using cell processes and cellular contractility. (E) Ligand density (shown as black bristles) affecting the extent of cell spreading. (F) Basement membrane composition: a slice of the basement membrane indicating key molecular components. (G) Fibrous ECM composition: a slice of fibrillar ECM listing several key components.

The ECM comprises predominantly protein and polysaccharide components (Frantz et al., 2010), but the forms of ECM can be remarkably diverse in biophysical, biochemical and topological properties (Naba et al., 2017; Shao et al., 2020). The precise composition of an ECM is often tissue specific, highly dynamic and responsible for its unique physical properties (e.g. topography, pore size, fiber size, fiber orientation, stiffness/elasticity and ligand density) (Fig. 2A-E) and chemical properties of each tissue (Brown, 2011; Dzamba and DeSimone, 2018; Hynes and Yamada, 2012). Although ECMs can exist in many forms, two major classes are basement membrane and interstitial ECM. Basement membranes are specialized, flat laminar ECMs consisting predominantly of core proteins organized into sheet-like networks of interconnected ECM molecules that include collagen IV, laminins and proteoglycans (e.g. perlecan) (Table 1; Fig. 2F) (Pozzi et al., 2017). Basement membranes underlie epithelia and surround the organs of most metazoans (Pozzi et al., 2017; Sekiguchi and Yamada, 2018). In interstitial matrices, collagens and various non-collagenous proteins (e.g. fibronectin, elastin, laminin and tenascin) contribute to the characteristic fibrous networks of ECMs, while proteoglycans and water contribute to their interstitial spaces (Table 1; Fig. 2G) (Frantz et al., 2010; Hynes and Yamada, 2012).

**Table 1.**
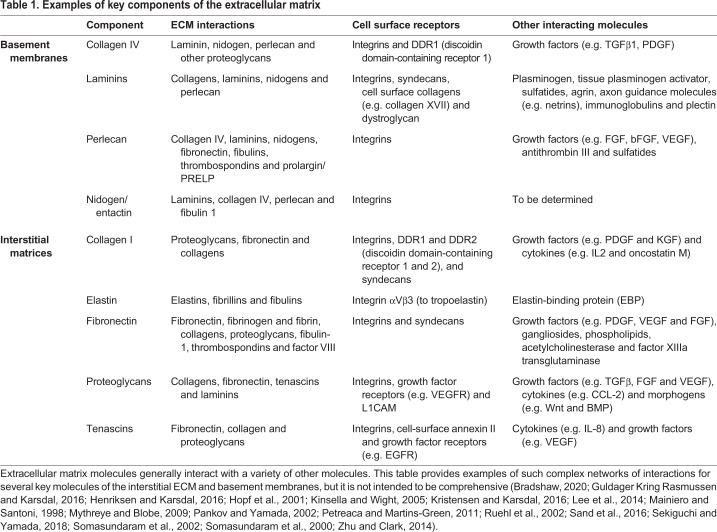
**Examples of key components of the extracellular matrix**

## Cell-ECM and cell-cell adhesions

Cell adhesions are the attachment structures between cells and the ECM, or between cells and other cells. They are essential for the organization of individual cells into three-dimensional tissues. The specific properties of cell-ECM adhesions, such as their distribution, quantity and stability/duration, can vary between organisms, tissues, developmental stages and even neighboring cells (Diaz-de-la-Loza et al., 2018; Etournay et al., 2015; Munster et al., 2019; Ray et al., 2015). Amidst this heterogeneity, the most common cell-ECM adhesions are mediated by integrins, which are linked to the internal cell cytoskeleton (Geiger et al., 2001). Cells use these adhesions to attach directly either to anchoring ligands of the ECM interstitial matrix (e.g. fibrous collagens, fibronectin and vitronectin) or to other glycoproteins in the basement membrane (e.g. laminin or network collagens) (Table 1; Fig. 1A) (Frantz et al., 2010; Hynes and Yamada, 2012). Indeed, several types of integrin-dependent adhesions are involved in crucial developmental processes (Alberts et al., 2014; Gillard et al., 2019; Keller, 2006). The best-characterized integrin-based adhesions are the RhoA-stimulated focal adhesions that anchor the ends of actin stress fibers to the nearby matrix. However, their precursors and variants (e.g. dot-like focal complexes and elongated fibrillar adhesions) are also likely to play roles in developmental events (Davidson et al., 2019; Goodwin et al., 2017; Horton et al., 2016a,b; Lee et al., 2018). Highlighting the importance of integrin-dependent adhesions in development, mutations in various integrin- and integrin-associated-protein family members are implicated in several human developmental diseases, such as those involving renal (Humbert et al., 2014), ocular (Beleggia et al., 2015; Zhang et al., 2016), pulmonary (Yalcin et al., 2015) and dermal/epidermal (Condrat et al., 2018; Mylonas et al., 2019) tissues.

Embryogenesis requires a coordinated balance between cell-ECM and cell-cell dynamics. Cell-cell adhesions mediate tissue cohesion and organization. Through cell-cell adhesions, the ECM can exert physical effects beyond only the first layer of ECM-attached cells into the interior of the tissue/organ. As with cell-ECM adhesions, a variety of cell-cell adhesive contacts are found in the developing embryo, for example various adherens junctions (Halbleib and Nelson, 2006; Letizia et al., 2019), desmosomes (Bharathan and Dickinson, 2019; Garrod and Chidgey, 2008) and tight junctions (Anderson and Van Itallie, 2009; Chan et al., 2019; Eckert and Fleming, 2008). Contacts mediated by the cadherin family of adhesion molecules are particularly important types of cell-cell interactions for maintaining organized solid tissues and transmitting mechanical signals (Balaji et al., 2019; Goodwin and Nelson, 2017; Halbleib and Nelson, 2006; Pinheiro and Bellaiche, 2018; Sumi et al., 2018; Wu and Taneyhill, 2019). As with integrin-mediated adhesions, human mutations in cadherin complexes are implicated in several developmental disorders (Accogli et al., 2019; Cox et al., 2018; Saeidian et al., 2019; Samuelov et al., 2015). For detailed descriptions of the diversity, physiological roles and biochemical properties of the numerous integrins, integrin-associated proteins, cadherins and adhesion complexes, we refer the reader to a number of recent excellent reviews (Bachmann et al., 2019; Green and Brown, 2019; Halbleib and Nelson, 2006; Horton et al., 2016a,b; Hynes, 1992; Hynes, 2002; Takeichi, 2014; Tepass et al., 2000).

In the context of this Review, the ECM uses cellular adhesions to regulate or modulate tissue shaping by anchoring, signal/force transmission and cell migration. Owing to the importance of cell migration in embryogenesis, we devote a separate section to this topic below.

### Tissue shaping by anchoring

**Fig. 3.**
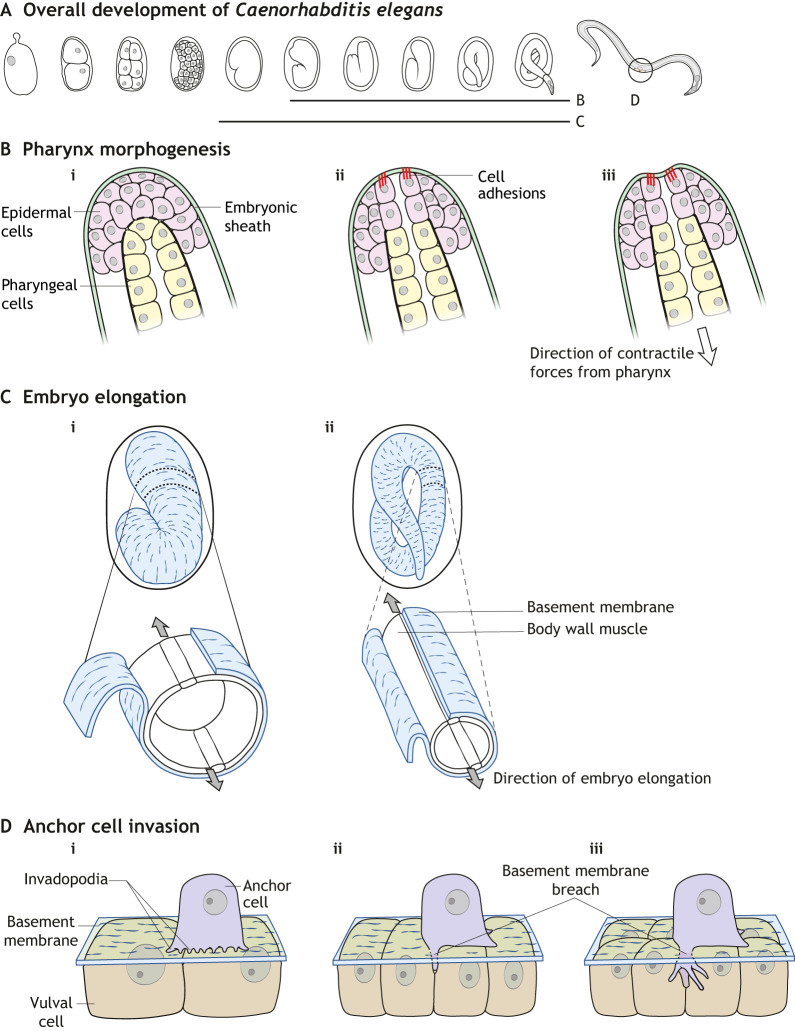
**Schematic diagrams of *C. elegans* model systems discussed in this Review.** (A) Overview of *C. elegans* development indicating stages involved in the following panels. (B) Pharynx morphogenesis. Epidermal cells adhering via cell adhesions to the surrounding embryonic sheath, which prevents deformation of the epidermis by pulling forces from the developing pharynx (pharyngeal cells in yellow). (C) Embryo elongation. The basement membrane serves as a ‘molecular corset’, acting in conjunction with muscle contractions to elongate the embryo. (D) Anchor cell invasion. Anchor cells use invadopodia to produce initial focal sites of basement membrane degradation (i). Upon breaching the basement membrane (ii), further invadopodia formation ceases, a large invasive protrusion forms and the anchor cell inserts itself between underlying vulval cells (iii).

Many developing organisms progress through stages in which a layer of ECM separates embryonic germ layers (Latimer and Jessen, 2010) and/or surrounds a mass of cells. For example, the vitelline envelope surrounds the blastoderm and oocyte of *Tribolium*, *Drosophila* and other non-mammalian species (Munster et al., 2019), the cuticle surrounds *Drosophila* larval and pupal tissues (Ray et al., 2015), and the zona pellucida surrounds the oocyte of humans and other mammals (Bhakta et al., 2019). In these systems, spatiotemporal variations in cell-ECM adhesions during tissue-intrinsic contraction drive tissue shaping. For example, the *Tribolium* integrin termed ‘inflated’ temporarily mediates adhesion of blastodermal cells to the antero-ventral region of the vitelline envelope. This localized attachment guides unidirectional tissue elongation, because myosin contractile activity causes the non-anchored dorsal tissues to slide along the envelope (Munster et al., 2019). Similarly, in *C. elegans*, the attachment of epidermal cells to an FBN-1 extracellular fiber meshwork of the embryonic sheath anchors the epidermis to prevent its posterior displacement secondary to pulling forces of the developing pharynx (Fig. 3A,B) (Kelley et al., 2015).

**Fig. 4.**
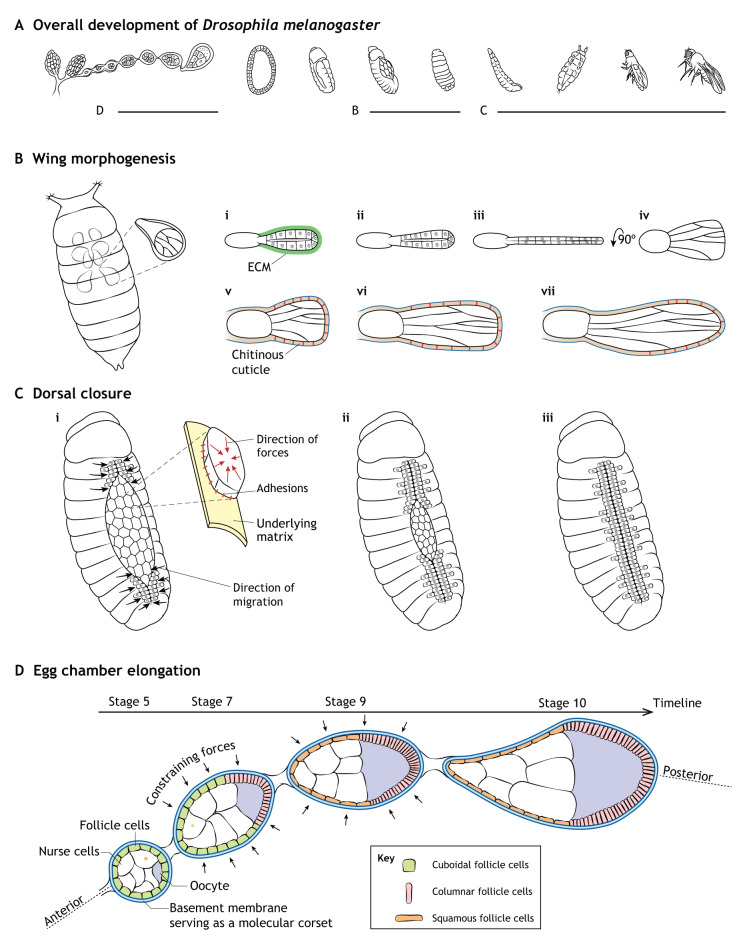
**Schematics of *Drosophila* model systems discussed in this Review.** (A) Overview of *Drosophila* development indicating stages involved in the following panels. (B) Wing morphogenesis. (i-iv) Removal of the ECM initiates wing elongation secondary to cell columnar-to-cuboidal shape changes. (v-vii) Dynamic patterned attachment of pupal wing epithelial cells to the chitinous cuticle shapes the developing wing. (C) Early (i), middle (ii) and late (iii) dorsal closure. Contracting cells adhering to underlying matrix along with lateral epidermal cells migrating towards the dorsal midline as the amniosera contracts and ingresses. (D) Egg chamber elongation. The basement membrane promotes cuboidal (green)-to-squamous (orange) transitions of anterior follicle cells and cuboidal-to-columnar (pink) transitions of posterior follicle cells; the basement membrane provides constraining forces as a ‘molecular corset’ to elongate the egg chamber.

Turning to insights provided by *Drosophila* systems, the apical ECM protein Dumpy (Dp) anchors distal epithelial cells of the *Drosophila* pupal wing to the surrounding chitinous cuticle in a patterned manner (Fig. 4A,B) (Ray et al., 2015). This Dp-mediated attachment resists tissue retraction that would otherwise result in the truncated wings, legs and antennae observed in *dp* loss-of-function mutants (Ray et al., 2015). Several *Drosophila* systems, including Dp-regulated limb morphogenesis, have been characterized by computational models that simulate the ability of cellular interactions to resist or transmit forces to drive oriented tissue growth during development (Etournay et al., 2015; Sui et al., 2018; Tozluoglu et al., 2019). In addition to force resistance and transmission, these cell-matrix interactions allow the ECM to dissipate forces exerted on cells during tissue morphogenesis. This buffering role of the ECM occurs during formation of the *Drosophila* leg disc (Proag et al., 2019). In early stages of this process, the peripodial epithelium remains in a relaxed state because tensile forces caused by leg elongation are borne by the attached ECM. At latter stages, however, cell-matrix interactions are lost, retractile forces are transferred to the cell monolayer and the peripodial epithelium opens and retracts (Proag et al., 2019). Embryogenesis requires cooperation between the physical cell-adhesion mechanisms discussed above and various signaling processes that transfer mechanical information between cells and tissues.

### Force and mechanical signal transmission

Appreciation of the roles of mechanical forces in developing tissues has grown from initial observations more than one century ago that documented load-induced bone remodeling (Churchill, 1970), to recent elaborate investigations using advanced biophysical techniques that include cell migration simulators, *in vivo* embryo remodeling quantification systems and others (Hou et al., 2019; Lardennois et al., 2019; Roca-Cusachs et al., 2017). The ability of a cell to sense and transduce mechanical signals (termed mechanosensation and mechanotransduction, respectively; Box 1) is fundamental to biophysically guiding tissue morphogenesis (Merle and Farge, 2018; Wozniak and Chen, 2009). Coordination of this signaling between cells and their physical environment during development depends on ECM biophysical properties (Fig. 2A-D) [e.g. geometry, alignment and elasticity (Humphries et al., 2017; Ma et al., 2013; Piotrowski-Daspit et al., 2017; Sopher et al., 2018; Yamada and Sixt, 2019)], cell-matrix adhesion (Fig. 1A) and intercellular adhesions.

**Box 1. Mechanotransduction and the ECM**

Cells not only synthesize and remodel the ECM, but also respond to mechanical information coming from the ECM. Cells sense physical stimuli from their microenvironment, such as ECM topography, composition and stiffness. These external signals are converted into cellular responses in the process of mechanotransduction. Research into the multiple mechanisms of mechanotransduction is rapidly expanding (reviewed by Chighizola et al., 2019; Doyle and Yamada, 2016; Jansen et al., 2017, 2015; Ringer et al., 2017).

The following are key terms in this rapidly evolving field:

- Mechanobiology: characterizing how cells and tissues respond to mechanical/physical stimuli through integrating biology, engineering and physics.
- Mechanosensitive: describing a molecule that undergoes structural changes in response to mechanical stimuli.
- Mechanosensing: the process of detecting mechanical stimuli.
- Mechanotransduction: the overall process of sensing a mechanical signal and converting it to an intracellular response.
- Mechanosignaling: intracellular signaling events induced by extracellular mechanical stimuli.

Among the wide variety of mechanisms of mechanotransduction [e.g. those involving cell-cell adherens junctions (Qin et al., 2020), mechanosensitive ion channels (Barzegari et al., 2020a,b; Jin et al., 2020) and others], integrin-based responses of cells to the extracellular matrix have recently been characterized in particular detail. As depicted schematically in a simplified form in Fig. 1A, the integrin cell-surface receptors for ECM molecules are heterodimers that bind to ECM molecules. Intracellular proteins bind to the cytoplasmic tails of integrins and organize multimolecular cell-matrix adhesion complexes in order to transmit external signals into the cell (Kechagia et al., 2019; Seetharaman and Etienne-Manneville, 2018; Sun et al., 2016). A number of excellent reviews discuss the molecular composition of integrin-associated and other force-stimulated (e.g. non-integrin focal adhesion, stretch-sensitive ion channel, etc.) protein complexes (Barzegari et al., 2020a,b; Doyle and Yamada, 2016; Jansen et al., 2015; Michael and Parsons, 2020).

Upon sensing mechanical stimuli, mechanosensitive molecules can initiate or modulate a wide variety of specific intracellular signaling pathways, including the Rho GTPase-related (Rafiq et al., 2019), Hippo (Barzegari et al., 2020a,b), TGFβ (Nguyen et al., 2020) and YAP/TAZ (Pocaterra et al., 2020) signaling pathways. This signaling can lead to diverse series of functional and/or structural responses in the involved cells and tissues – the most extensively investigated of these are highlighted in each section of this Review (e.g. shape changes, migratory events and differentiation) (Fig. 1).

For example, *Drosophila* dorsal closure relies on integrin-mediated cell-matrix adhesions for the transmission of intercellular tensile forces generated by cell constriction (Goodwin et al., 2016). When the number of these integrin-mediated focal adhesion-like structures is modified, intercellular apical force transmission is perturbed. The resulting abnormal contraction and ingression of the amniosera prevents opposing embryonic lateral epidermal cells from normally migrating toward the dorsal midline (Fig. 4C) (Goodwin et al., 2016). Besides these integrin-mediated cell-ECM adhesions, cadherin-mediated cell-cell adhesions are also crucial for proper force transmission across cells that make up the amniosera. Changes in cadherin localization and stability result in a similar failure of dorsal closure (Goodwin et al., 2017). In fact, cell-cell and cell-matrix adhesions functionally interact to promote proper mechanical signaling in these developing tissues (Goodwin et al., 2016, 2017).

Many morphogenetic events in embryogenesis are coordinated by muscle contractions, such as embryo elongation in *C. elegans* (Fig. 3C) and skeletogenesis in zebrafish, mouse, chick and other vertebrates (Shwartz et al., 2012). During these processes, mechanical information from muscle fibers is relayed to local environments via matrix-muscle adhesions and transmitted throughout tissues by cell-matrix and cell-cell junctions. Moreover, elongating *C. elegans* embryos rely on the ECM to coordinate communication between muscle, lateral epidermal and dorsal/ventral epidermal tissues (Gillard et al., 2019). In this system, mechanical signals generated by muscle contractions are transmitted to and between epidermal cells via cell-matrix molecular tendons and adherens junctions, respectively. When functional mutations alter matrix-muscle adhesion proteins (e.g. altered NOAH-1 and NOAH-2), muscle contractions cannot convey signals through molecular tendons, intracellular actin fibers fail to polarize, cells orient inadequately along the anterior-posterior axis of the embryo and mid-elongation arrest ensues (Vuong-Brender et al., 2016, 2017). These studies, together with others investigating cell adhesion complexes in developing tissues, indicate that cell-matrix interactions can regulate tissue shape using anchoring by relaying mechanical signals between cells and tissues, and by coordinating migratory events.

## Migration

Cell migration is crucial for embryogenesis: cells undergo initial specification during gastrulation and can then migrate separately or as collective assemblies guided by environmental cues to reach their destinations (Friedl and Gilmour, 2009; Scarpa and Mayor, 2016; Yamada and Sixt, 2019). These cues can be biochemical, such as diffusible or substrate-bound ligands (known as chemotaxis or haptotaxis, respectively) or physical, mediated by substrate composition, topography (e.g. contact guidance) and stiffness (durotaxis), which can regulate migration and differentiation (Fig. 1B,D; Fig. 2). Advances in live-cell/tissue imaging, tunable biomaterials and *in vitro* models have revealed mechanisms through which the ECM can regulate a large repertoire of cell migration modalities (Feng et al., 2019; Li et al., 2017; Trappmann et al., 2017; Wang et al., 2019a,b; Yamada et al., 2019; Yamada and Sixt, 2019). In developing tissues, the ECM provides paths that can provide both directional and stop signals for coordinating cell migration.

Numerous model systems have provided insights into cell migration during embryogenesis (Scarpa and Mayor, 2016). Among these, studies of neural crest cell (NCC) development have substantially enhanced our understanding of complex ECM-cell interactions that govern migration. Triggered by cues such as substrate stiffness changes and transcription factors (Barriga et al., 2018; Hockman et al., 2019; Shellard and Mayor, 2019), NCCs display multiple migration modes [e.g. as organized groups, chains, sheets and/or relatively unorganized masses (Rozario and DeSimone, 2010; Shellard and Mayor, 2019; Theveneau and Mayor, 2011)]. In addition, NCC migration is regulated through mechanisms that include contact inhibition of migration (Bahm et al., 2017; Li et al., 2019b; Roycroft et al., 2018; Yoon et al., 2018), durotaxis (Barriga et al., 2018; Chevalier et al., 2016), and chemotaxis (Bajanca et al., 2019; Shellard et al., 2018; Szabo and Mayor, 2018).

Several recent reviews discuss coordinated ECM-NCC interactions during NCC migration in chick, mouse and *Xenopus* systems (Kechagia et al., 2019; Szabo and Mayor, 2018; Yamada and Sixt, 2019). We instead focus on recent studies and concepts discussing how ECM-cell interactions drive tissue formation in other systems, such as cells of the zebrafish ectoderm and mesendoderm, *Xenopus* mesendoderm and neurons and *Drosophila* myotubes, as well as in embryonic cell invasion.

### Roads and maps

Embryonic cell migration can include amoeboid, mesenchymal or lobopodial 3D modes of cell migration [the multiple modes of 3D cell migration are reviewed by Yamada and Sixt (2019)]. Mesenchymal and lobopodial migration involve extensive integrin-mediated adhesion to surrounding ECM substrates, whereas amoeboid migration can involve non-specific interactions with ECM. Mesenchymal migration is characterized by cells using actin-driven lamellipodial or filopodial protrusions to adhere to, produce force against and migrate in or on the ECM (Caswell and Zech, 2018; Ghiglione et al., 2018; Plutoni et al., 2019; Sharma et al., 2018; Zeledon et al., 2019). ECM biochemical and physical properties can regulate these leading edge protrusions and the resulting directed locomotion (Love et al., 2018; Plutoni et al., 2019).

The interplay between ECM constituents and cellular migration machinery is remarkably complex, requiring various membrane-bound and secreted cellular proteins to interact functionally at the cell-ECM interface to mediate or modulate embryonic cell migration (Bjerke et al., 2014; Cheng et al., 2019; Sánchez-Sánchez et al., 2017). As portrayed in the following paragraphs, the identity and quantity of expressed proteins determine the positions of cell protrusions, the dimensions and stability of cell-ECM interfaces, and the ability of cells to sense and respond to their microenvironments.

An early step in organized migration is the formation of oriented protrusions. For example, zebrafish prechordal plate cells secrete Cthrc1a (collagen triple helix repeat containing 1a) to generate polarized protrusions that interact functionally with fibronectin, and undergo extensive directed cell migration during axis extension and head formation (Cheng et al., 2019). Depletion of *cthrc1a* results in failed epiboly, diminished anteroposterior axis elongation and head defects (Cheng et al., 2019). Furthering this concept, ectodermal and mesodermal cells express the membrane-bound planar cell polarity protein Vangl2 (Vang-like 2) to form oriented actin-rich protrusions, achieve proper mediolateral alignment and elongation, and establish planar cell polarity in the zebrafish gastrula. Migrating *vangl2* mutant cells lack directionality, but increasing the fibronectin content of the surrounding ECM can repolarize the large protrusions of *vangl2* mutants and restore directional migration (Love et al., 2018).

The stability and dimensionality of cell-ECM interfaces influence cell migration. *Drosophila* embryonic hemocytes require prolonged, stable cell-ECM interactions to migrate along the ventral nerve cord. Hemocytes achieve stability of lamellipodia and prolonged cell-ECM adhesion states through autocrine deposition of laminin, a major basement membrane structural glycoprotein, in a Rab8-regulated manner (Sánchez-Sánchez et al., 2017). Exemplifying the importance of cell-ECM adhesive contact area, *Xenopus* mesendodermal cells increase expression of focal adhesion kinase during directional migration (Bjerke et al., 2014; Hens and DeSimone, 1995). Antisense morpholino oligonucleotide knockdown of focal adhesion kinase reduces the area of focal adhesion contacts, causes aberrant actin organization and uncoordinated cell protrusions, and, most notably, reduces spreading/traction forces and migration speed (Bjerke et al., 2014). At the tissue level, this focal adhesion kinase reduction results in defective neurulation, axial elongation and somitogenesis (Bjerke et al., 2014).

To reach their destination and circumvent and/or break down potential ECM barriers, cells must sense and react to their microenvironment. Recent reviews describe how mechanosensitive retinal ganglion cell axons in the developing *Xenopus* brain traffic along stiffness gradients to achieve proper anatomic distribution (Buchsbaum and Cappello, 2019; Long and Huttner, 2019). The concept of ECM stiffness and viscoelasticity regulating cell migration has been characterized in a variety of NCC systems (Barriga et al., 2018; Barriga and Mayor, 2019; Chaudhuri et al., 2015; Wang et al., 2019a).

### Stop signs and road blocks

Recent publications illustrate the intricate ECM-cell exchanges of information that can either prevent initiation of migration or ensure that migrating cells halt at their intended destination. Common mechanisms through which the ECM regulates these events include providing zones of uniform cytokine/growth factor concentration in place of gradients during chemotaxis or haptotaxis (Colak-Champollion et al., 2019; Malhotra et al., 2018; Sieg et al., 2000), forming physical barriers to restrict cell movement (Renkawitz et al., 2019; Zanotelli et al., 2019), and providing signaling cues to alter the cellular machinery responsible for protrusion, adhesion and/or traction force generation (Richier et al., 2018; Sekine et al., 2012; Sieg et al., 2000; Yamada and Sixt, 2019). These ECM signals cooperate with other forms of signaling during development.

A well-characterized example of attractant-guided cell migration involves the Cxcl12-Cxcr4/Cxcr7 signaling pathway. During embryogenesis, migrating mouse, chick, zebrafish and human primordial cells express the chemokine receptor Cxcr4. Stationary somatic cells express both the chemoattractant Cxcl12 to guide trafficking primordial cells, as well as the chemokine receptor Cxcr7 to endocytose excess Cxcl12 (Boldajipour et al., 2008; Breau et al., 2012; Colak-Champollion et al., 2019; Dalle Nogare et al., 2014; Friedl and Gilmour, 2009; Lei et al., 2019; Neelathi et al., 2018; Zheng et al., 2018). In these systems, the concentration of Cxcl12 serves as either a ‘green light’ or a ‘stop sign’ for migrating primordial cells. This chemokine signaling pathway regulates several cellular events not only in development, but also in disease (Del Molino Del Barrio et al., 2018; Pluchino et al., 2018; Teicher and Fricker, 2010; Zheng et al., 2019). In addition, numerous cytokines/chemokines regulate processes in cell migration and embryogenesis that are beyond the scope of this Review, but are discussed elsewhere (Devreotes and Horwitz, 2015; Haeger et al., 2015).

To navigate through physical barriers, cells remodel either their cytoskeleton or the surrounding ECM. The cell nucleus is a relatively large and stiff cytoplasmic organelle that limits the capacity of migrating cells to squeeze through barriers, such as ECM pores that can serve as road blocks to migrating cells (Fig. 2B) (Denais et al., 2016; Harada et al., 2014; Yamada and Sixt, 2019). The structural proteins lamins A and C are major contributors to nucleoskeletal stiffness, and their expression correlates with the ability of a cell to navigate through spaces and pores in the ECM (Bone and Starr, 2016; Chen et al., 2018a,b; Das et al., 2019; Harada et al., 2014; Renkawitz et al., 2019). Alternatively, cells can either proteolytically or non-proteolytically deform their microenvironment (Gifford and Itoh, 2019; van Helvert et al., 2018; Wang et al., 2019c; Wolf and Friedl, 2011). This proteolytic mechanism is nicely portrayed by dorsally migrating endodermal cells during zebrafish gastrulation (Hu et al., 2018). These cells regulate Mmp14a/b (matrix metalloproteinase 14) levels through expression of Gpc4 (glypican 4). In *gpc4* zebrafish mutants, loss of functional Gpc4 impairs cell migration due to increased amounts of ECM fibronectin and laminin caused by diminished proteolytic degradation (Hu et al., 2018).

A particularly important determinant of cell trafficking is the presence and activation of functional cellular migration machinery. For example, modifying the cellular contractile apparatus can have an even greater effect on cell migration than altering the surrounding ECM microenvironment, as demonstrated during contact guidance of cells migrating in 3D collagen matrices (Nuhn et al., 2018). The complex interplay between signaling, adhesions and matrix assembly is exemplified by the transcription factor Pitx2 (paired-like homeodomain 2) and its downstream activities. Classically characterized by its involvement in left-right patterning during asymmetric morphogenesis, recent insights suggest that Pitx2c serves an additional key role in chemokine-ECM-integrin-dependent mesendodermal migration in early embryogenesis (Collins et al., 2018). Using *pitx2c*-deletion mutant zebrafish embryos, Pitx2c expression has been shown to promote mesendodermal cell migration by coordinating Cxcl12b chemokine signaling, integrin β1 expression and ECM fibronectin assembly (Collins et al., 2018). Pitx2 is not only crucial for zebrafish embryogenesis, but also for mouse (Mitiku and Baker, 2007), *Xenopus* (Ding et al., 2017), chicken (Torlopp et al., 2014) and human development (Hendee et al., 2018; Yin et al., 2014; Zhang et al., 2019).

Further demonstrating the ability of the ECM to modify the cellular migration machinery, ECM cues can actively suppress sensory actin-rich filipodia in an integrin-dependent manner (Richier et al., 2018). This role is observed in elongating *Drosophila* myotube tips that probe the ECM to locate ‘stop signs’ (the matrix overlying tendon cells) and establish sites of tendon attachment during lateral transverse muscle development. Exploratory and sensory behavior of cellular protrusions, cell-substrate adhesion and cell traction-force generation involve a multitude of signaling mechanisms that contribute to cell migration during embryogenesis (Devreotes and Horwitz, 2015; Doyle and Yamada, 2016; Lauffenburger and Horwitz, 1996; Ridley et al., 2003).

Conversely, a cell that is initially restrained by a barrier such as the underlying basement membrane can use multiple strategies to breach it. A particularly striking developmental example – reminiscent of human cancer cell invasion – is used by the *C. elegans* anchor cell for vulval invasion (Fig. 3D). This cell initially produces focal points of degradation of the basement membrane using invadopodia, which then proceeds to a large breach using a combination of matrix metalloproteinase degradation and forces generated by actin polymerization driven by Arp2/3 (Cáceres et al., 2018; Kelley et al., 2019; Naegeli et al., 2017). Interestingly, even if protease function is inhibited, the local deformation forces fueled by local mitochondrial enrichment and expanded by lysosomal fusion to form a large protrusion are ultimately sufficient to breach the basement membrane barrier to permit invasion (Sherwood and Plastino, 2018).

## Morphology and polarity

In addition to its well-known role as a scaffold (Frantz et al., 2010) the ECM can regulate morphological properties of cells and tissues via a variety of mechanical cues. Cells drive morphogenesis through a series of changes in three-dimensional shape (Fig. 1C), orientation and position [e.g. columnar-to-cuboidal (Diaz-de-la-Loza et al., 2018), cuboidal-to-columnar (Balaji et al., 2019), polarity, intercalation (Chen et al., 2019), etc.] to provide a diverse toolbox for shaping the developing embryo.

The ECM uses this toolbox to help coordinate development. Matrix physical properties, e.g. stiffness, elasticity, density and fiber orientation (Fig. 2) (Chen et al., 2019; Chlasta et al., 2017; Diaz-de-la-Loza et al., 2018), influence local cell and tissue shape and polarity.

### Flattening

Recent advances in 3D cell culture techniques have revealed how cell shape changes can drive morphogenesis (Diaz-de-la-Loza et al., 2018; Doyle and Yamada, 2016; Yamada and Sixt, 2019). Cell shape change, along with oriented cell division (Godard and Heisenberg, 2019) and polarized cell intercalation (Huebner and Wallingford, 2018), contribute to driving epithelial elongation in development.

In *Drosophila* wing and leg elongation, after the peripodial layer is removed, ECM remodeling is responsible for initiating wing elongation (Fig. 4B). Triggered by this matrix remodeling, neighboring cells flatten (completing a columnar-to-cuboidal transition) to drive lateral tissue expansion (Diaz-de-la-Loza et al., 2018).

Furthermore, both cell and tissue shape are influenced by mechanical properties of the basement membrane in the developing *Drosophila* follicle (Chlasta et al., 2017). A TGFβ-driven decrease in basement membrane stiffness is associated with flattening of anterior follicle cells, which contributes to the final elongated morphology of the egg chamber (Fig. 4D) (Chlasta et al., 2017). In other species, several investigations have identified that similar cell flattening events are responsible for embryonic morphogenetic changes in zebrafish (Bruce, 2016; Dasgupta et al., 2018; Delile et al., 2017) and *Xenopus* (Kloc and Kubiak, 2014).

### Constraining

While the ECM promotes flattening of the anterior *Drosophila* follicle cells, it simultaneously constrains the posterior follicle cells to induce a cuboidal-to-columnar shape transition (Balaji et al., 2019; Chlasta et al., 2017). Specifically, between stages 6 and 9 of egg chamber development, the basement membrane physically constrains the underlying cells at the posterior pole. In conjunction with medial myosin II contraction and adherens junction remodeling, follicle cells undergo a resulting cuboidal-to-columnar transition (Fig. 4D) (Balaji et al., 2019).

Beyond its effects on individual cells, fibrillar structures of the ECM provide anisotropic constraining forces to drive and orient morphogenetic events at the tissue level (Isabella and Horne-Badovinac, 2016; Vuong-Brender et al., 2017). This is classically illustrated by the polarized fibrillar basement membrane serving as a ‘molecular corset’ surrounding the growing *Drosophila* egg chamber (Gutzeit et al., 1991; Isabella and Horne-Badovinac, 2016; Ramos-Lewis and Page-McCaw, 2019). In this case, the basement membrane physically constrains outward expansion of the egg chamber to force growth to occur along the anterior-posterior axis (Fig. 4D) (Chen et al., 2019; Isabella and Horne-Badovinac, 2016; Ramos-Lewis and Page-McCaw, 2019). A similar phenomenon is observed in *C. elegans*, in which the ECM not only constrains the shape of the embryo, but also provides crucial attachment sites for contracting muscle fibers (Fig. 3C) (Vuong-Brender et al., 2016, 2017).

### Polarizing

The ECM provides information regulating cell orientation and polarity. For example, stiffness cues provided by the basement membrane of the developing *Drosophila* follicle regulate polarized reorientation of anterior follicle cells (Chen et al., 2019). When these cues are compromised, Src tyrosine kinase-driven remodeling of cell-cell junctions is altered, anterior follicle cells randomly orient along the anterior-posterior axis and the organ fails to achieve its appropriate shape (Chen et al., 2019).

Polarity and orientation of cells and tissues is closely regulated by several factors, including adhesion complexes, actin organization, actomyosin contraction and ECM signals (Gillard et al., 2019). For example, the ECM surrounding the elongating *C. elegans* embryo is essential for establishing bipolar planar polarity of the apical PAR module (a protein complex responsible for organizing cell junctions at the apical cell surface) of lateral epidermal cells (Gillard et al., 2019). The resulting planar organization of actin helps to orient cell-shape changes and polarize the developing embryo. Indeed, genetic depletion of the ECM protein perlecan results in altered actin planar polarity and cell orientation (Gillard et al., 2019).

Many of the investigations characterizing the ability of the ECM to regulate polarity and orientation in development are limited to the *Drosophila* and *C. elegans* models, as described in this section. This is probably because of the complexity of comprehensively analyzing 3D *in vivo* embryogenic events in mammals (Chan et al., 2017; Herrera-Perez and Kasza, 2019; Shahbazi et al., 2019). Investigations in this field may soon rapidly expand as emerging techniques provide the ability to manipulate *in vivo* mechanical signals directly in the developing embryo (Chan et al., 2017; Stooke-Vaughan and Campàs, 2018).

## Differentiation

Specific ECM microenvironmental niches, biochemical cues and mechanical signals are intriguing candidate factors for guiding the differentiation of pluripotent embryonic stem cells or induced pluripotent stem cells, as well as fate-restricted adult stem cells (e.g. mesenchymal, hematopoietic, neural or epithelial) (Harvey et al., 2019; Liu et al., 2019; Smith et al., 2018; Zhu and Huangfu, 2013). The ECM regulates stem cells through a complex mixture of mechanical cues that include matrix geometry and chirality (Chen et al., 2018a; Dong et al., 2019; von Erlach et al., 2018; Wei et al., 2019), rigidity (Gerardo et al., 2019), ligand density (Lee et al., 2019) and topography (Fig. 2) (Abagnale et al., 2015; Hulshof et al., 2017). A number of recent reviews have addressed the broad topic of ECM mechanical properties that can regulate stem cell fate (Kumar et al., 2017; Kumari et al., 2018; Smith et al., 2018; Vining and Mooney, 2017). Consequently, this Review provides a brief overview of key concepts in this field and refers readers to recent relevant literature for additional information.

### Stem cell fate specification

In addition to promoting stem cell support within a specific microenvironmental niche, the mechanical properties of the ECM can strongly influence a variety of stem cell behaviors including maintenance, self-renewal, proliferation and differentiation (Fig. 1D) (Gattazzo et al., 2014). For example, manipulating substrate elasticity/stiffness alters mesenchymal stem cell (MSC) fate (Darnell et al., 2018a,b; Gerardo et al., 2019; Lee et al., 2019; Engler et al., 2006) and embryonic stem cell (ESC) fate (Przybyla et al., 2016), driving cell lineage commitment towards tissues with similar physical properties, e.g. towards soft adipose or stiff osseous tissue. ECM topographical cues, including geometric chirality (Dong et al., 2019; Wei et al., 2019) and ligand density (Fig. 2E) (Darnell et al., 2018a,b; Lee et al., 2019), as well as matrix stress-relaxation cues (Darnell et al., 2018a,b), influence MSC differentiation. For example, transcriptional changes occur in mouse MSCs when cultured on substrates of varying adhesion ligand density, stiffness and stress relaxation rate, which drive osteogenic or hematopoietic differentiation (Darnell et al., 2018a,b). Similar studies with ESCs characterize the role of surface roughness of the substrate (Jaggy et al., 2015) and the size of landscape features (Fig. 2A) (Lapointe et al., 2013; Macgregor et al., 2017; Reimer et al., 2016) in modulating ESC differentiation potential. Various molecular pathways (e.g. Notch and Wnt/β-catenin signaling, among others) are hypothesized to serve prominent roles in regulating the responses of stem cells to substrate mechanics (Harvey et al., 2019; Kumari et al., 2018; Przybyla et al., 2016; Smith et al., 2018; Totaro et al., 2017). Consequently, the biophysical properties of the ECM regulate stem cell differentiation through a coordinated balance of multiple physical mechanisms, the complexity and mechanisms of which are only starting to be characterized (Harvey et al., 2019; Muncie and Weaver, 2018; Smith et al., 2018; Wen et al., 2014).

### Potential applications

Compared with adult stem cells, the body of literature describing biophysical contributions of the ECM to pluripotent ESC differentiation for potential clinical application is currently strikingly sparse (Kumari et al., 2018), with many investigations focusing on the topographical features described above. Such topographical studies move us closer to possible future use of ESCs in clinical tissue regeneration, but a complicating feature is that ESCs are known to respond to a wide range of both biomechanical and biochemical cues (Dogan, 2018). Consequently, even though some adult stem cells, such as hematopoietic stem cells, have achieved clear success in regenerative medicine (Iida et al., 2019), the clinical use of ESCs remains controversial for both ethical and practical reasons (Prentice, 2019). To further our understanding of embryonic development and to continue making progress towards potential clinical applications of embryonic stem cells, the complex interplay between the ECM and ESCs should be better characterized in terms of ECM roles and mechanisms for providing specific microenvironmental niches and biomechanical regulatory mechanisms that can guide cell fate.

## Conclusions and future perspectives

Over the past decade, classical and innovative research approaches and techniques (Box 2) have identified many diverse biophysical and mechanical roles for the ECM during morphogenesis of many organisms and model systems, including zebrafish, *Drosophila*, *C. elegans* and *Xenopus*. Considerably less is known about the biophysical regulation of embryos developing *in utero*, although *in ovo* studies in avian embryos (Gandhi and Bronner, 2018) and new intrauterine methods for mammalian embryos (Beronja et al., 2010; Iwashita et al., 2014) are providing new opportunities to overcome the technical problems of smaller sample size, inaccessibility and long gestational periods.

**Box 2. Techniques for analyzing roles of ECM in development**

- Gene ablation and overexpression altering ECM composition (George et al., 1993; Liu et al., 2020; Oh et al., 2013; Schinzel et al., 2019; Terajima et al., 2019; Wang et al., 2019c)
- Crosslinking of ECM constituents (Deo et al., 2020; Petrie et al., 2012; Piersma et al., 2020; Vallet and Ricard-Blum, 2019)
- Alteration in alignment, pore size or other physical parameters (Paul et al., 2019; Wolf et al., 2013; Yamada and Sixt, 2019)
- Experimentally induced individual protein degradation (Cavanaugh et al., 2020; Li et al., 2019a; Reynders et al., 2020)
- Specific antibodies, pharmacological agents and other inhibitors (Afasizheva et al., 2016; Kapoor et al., 2020; Lu et al., 2020; Valiente-Alandi et al., 2018)
- Optogenetic activation or depletion of proteins (Baaske et al., 2019; Liu et al., 2016; Reynders et al., 2020)
- 3D tissue and organ culture (Clevers, 2016; Yamada and Cukierman, 2007)
- Atomic force microscopy (AFM) and microrheology (Alcaraz et al., 2018; Staunton et al., 2019; Viji Babu et al., 2019)
- Laser ablation (Balcioglu et al., 2016; Goodwin et al., 2016; Ilina et al., 2011)
- Force-sensing molecules [e.g. chimeras with vinculin, talin or peptides (Brockman et al., 2018; Cost et al., 2019; Curry et al., 2018; Grashoff et al., 2010; Rothenberg et al., 2018)]
- Local force determination via analyzing droplet deformation (Campas et al., 2014; Serwane et al., 2017)
- Local application of force [e.g. by magnetic beads or optical tweezers (Herath et al., 2014; Honarmandi et al., 2011; Jones et al., 2015; Roca-Cusachs et al., 2009)]

Adding to the complexity of species differences in development, the ECM is not static during embryogenesis. Developing organs and tissues interact with similarly dynamically changing matrices throughout embryogenesis (Loganathan et al., 2016). To address some of these hurdles, *in vitro* bioengineered models recapitulating key milestones of mammalian development provide preliminary insights into the mechanisms of ECM mechanical regulation (Vianello and Lutolf, 2019). However, such models merely skim the surface of the intricate mechanical and molecular signaling systems in embryogenesis.

What controls the changing biophysical properties of the ECM at progressive developmental stages? What molecular mechanisms allow the ECM and the cells that synthesize ECM to sense and respond to cues from cells and tissues? Are these mechanisms consistent between different tissue types? Are such mechanisms conserved between different organisms? These and many other unanswered questions, combined with rapidly emerging new techniques to explore these topics, make this an exciting time for the field of ECM developmental biology. The field is likely to move toward increasingly quantitative approaches involving directly quantifying changing ECM composition and physical parameters as development proceeds, combined with mathematical modeling to characterize mechanisms and generate new testable hypotheses. ECM molecules continue to be identified as therapeutic and prognostic targets in disease (Theocharis et al., 2019). Approaches to precisely control synthetic ECM forces and properties (van Oosten et al., 2019; Wu et al., 2018) are emerging and tissue engineering strategies focusing on biophysical properties of the ECM (Petersen et al., 2018) are rapidly progressing. Novel methods to produce completely autologous implants (Edri et al., 2019) are being explored. Besides ultimately gaining a satisfyingly deep mechanistic understanding of the roles of ECM in development, we can hope to begin to link the basic biophysics of ECM embryology to the clinical field of regenerative medicine.
